# Autoimmunity in gestational diabetes mellitus in Sardinia: a preliminary case-control report

**DOI:** 10.1186/1477-7827-6-24

**Published:** 2008-06-29

**Authors:** Cinzia Murgia, Marisa Orrù, Elaine Portoghese, Nicoletta Garau, Pierina Zedda, Rachele Berria, Costantino Motzo, Simonetta Sulis, Michela Murenu, Anna Maria Paoletti, Gian Benedetto Melis

**Affiliations:** 1 Dipartimento Chirurgico Materno Infantile e di Scienza delle Immagini, Sezione di Clinica Ginecologica, Ostetrica e Fisiopatologia della Riproduzione Umana, Universita' degli Studi di Cagliari, Italy; 2 Department of Obstetrics and Gynecology, Case Western Reserve University, 44109 Cleveland, Ohio, USA

## Abstract

**Background:**

We previously reported a high prevalence (22.3%) of gestational diabetes mellitus (GDM) in a large group of Sardinian women, in contrast with the prevalence of Type 2 diabetes. Sardinia has an unusual distribution of haplotypes and genotypes, with the highest population frequency of HLA DR3 in the world, and after Finland, the highest prevalence of Type 1 diabetes and Autoimmune-related Diseases. In this study we preliminarily tested the prevalence of serological markers of Type 1 diabetes in a group of Sardinian GDM patients.

**Methods:**

We determined glutamic decarboxylase antibodies (anti-GAD65), protein tyrosine phosphatase ICA 512 (IA2) antibodies (anti-IA2), and IAA in 62 GDM patients, and in 56 controls with matching age, gestational age and parity.

**Results:**

We found a high prevalence and very unusual distribution of antibodies in GDM patients (38.8%), the anti-IA2 being the most frequent antibody. Out of all our GDM patients, 38.8% (24 of 62) were positive for at least one antibody. Anti-IA2 was present in 29.0 % (18 out of 62) vs. 7.1% (4 out of 56) in the controls (P < 0.001). IAA was present in 14.5% (9 out of 62) of our GDM patients, and absent in the control subjects (P < 0.001). Anti-GAD65 was also present in GDM patients, with a prevalence of 3.2% (2 out of 62) while it was absent in the control group (P = NS). Pre-gestational weight was significantly lower (57.78 ± 9.8 vs 65.9 ± 17.3 *P *= 0.04) in auto-antibodies- positive GDM patients.

**Conclusion:**

These results are in contrast with the very low prevalence of all antibodies reported in Italy. If confirmed, they could indicate that a large proportion of GDM patients in Sardinia have an autoimmune origin, in accordance with the high prevalence of Type 1 diabetes.

## Background

Gestational diabetes mellitus (GDM) is defined as "carbohydrate intolerance of variable severity with onset or first recognition during pregnancy" [1] and affects 1–14% of all pregnancies, depending on the population studied, the diagnostic test and its glycemic cut-off. Its prevalence mirrors that of Type 2 diabetes mellitus [2,3].

The prevalence of GDM in Italy was reported to be 2.3–10% [4,5]. A recent study of ours found [6] a surprisingly high prevalence (22.3%) of GDM in a large group of Sardinian women, in contrast with the prevalence of Type 2 diabetes in Sardinia. In fact, the prevalence of Type 2 diabetes in Sardinia is similar to that of other non high risk populations, while after Finland, it has the highest prevalence in the world of Type 1 diabetes mellitus and Type 1 diabetes- related Autoimmune Diseases, such as Multiple Sclerosis, Celiac Disease, Autoimmune Thyroid Disease [7-11]. Compared to other Caucasian populations Sardinia has an unusual distribution of haplotypes and genotypes, with the highest population frequency of HLA DR3 in the world, which partially explains the high incidence of Type 1 diabetes [12,13]. For these reasons Sardinia is an ideal population to study environmental, genetic and immunological factors involved in the pathogenesis of different diseases.

Type 1 diabetes results from a chronic autoimmune destruction of the insulin-secreting pancreatic beta cells, probably initiated by exposure of a genetically susceptible host to an environmental agent. During the preclinical phase, this autoimmune process is marked by circulating auto-antibodies against pancreatic islets or against beta cell antigens, such as islet cell antibodies (ICA), glutamic decarboxylase antibodies (GADA, recently replaced by the anti-GAD65, more specific for Type 1 diabetes), protein tyrosine phosphatase ICA 512 (IA2) antibodies (anti-IA2), and auto-antibodies to Insulin (IAA). These auto-antibodies (Diabetes-Related Auto-antibodies, DRAs) are present years before the onset of Type 1 diabetes and prior to any clinical symptoms. Preliminary studies have shown that the progression of Type 1 diabetes in Sardinia is also accompanied by an increased frequency of a combination of ICA with GAD or IA2 antibodies, or both [14].

A variable percentage of women with GDM are reported to be positive for the DRAs [15-23]. In these patients gestational diabetes is caused by the destruction of β-pancreatic cells by an auto-immune process as a result of interaction between genetic and environmental factors, in a similar way to what occurs in Type 1 diabetes, which leads to an insulin deficiency. The prevalence of DARs usually mirrors the prevalence of Type 1 diabetes outside pregnancy.

The prevalence of GAD antibodies in GDM patients has been shown to range between 0 and 38 %, that of ICAs between 1 and 38 %, that of IAA between 0 and 18%, and that of anti-IA2 between 0 and 6.2%. In Italy the prevalence of DARs in GDM patients has been reported to be very low [18,19]. Besides the different methods of study and laboratory procedures employed, the heterogeneity of the results is due to the different genetic and environmental background of each population, which determines the susceptibility to auto-immunity. Today auto-immune GDM is considered a heterogeneous disorder, which may result in various types of expression of the immune reactivity against the β-cells [23].

In women with GDM DRAs have been found to have a high positive predictive value for Type 1 diabetes after pregnancy [20-22,24]. GDM patients with positivity for DRAs can rapidly develop Type 1 diabetes after pregnancy and have peculiar characteristics. They are often slim, younger that those who develop Type 2 diabetes and have, as in Type 1 diabetes, an increased prevalence of HLA DR3 or DR4-DQ8 haplotypes. In addition, the majority of these women require insulin to treat their diabetes during pregnancy [21,24].

The frequency of DRAs in Sardinian women with GDM is still unknown. Considering the peculiarity of the Sardinian population, the high prevalence of Type 1 diabetes and the high prevalence of GDM on the island, it may be theorize that a large proportion of our GDM could have an autoimmune origin. Such a hypothesis needs to be confirmed by a large study assessing the prevalence of DRAs in a large GDM population, and by a follow-up study to verify their predictive value for the development of Type 1 diabetes. Nevertheless, on these grounds, we attempted to test this hypothesis in a pilot study.

## Methods

Anti-GAD65, anti-IA2, and IAA were determined in 62 GDM patients and in 56 controls with matching age, gestational age and parity, consecutively referred to our Obstetric Diagnostic Centre. The purpose and nature of the study were explained to all subjects, and their consent was obtained before their participation. The local Institutional Review Board approved the study.

All patients were Sardinian of Sardinian descent. None of them had a history of GDM, Type 1 or Type 2 diabetes. Diagnosis of GDM was made by a 100-g Oral Glucose Tolerance Test (OGTT) on the ADA's recommendation (Carpenter and Coustan criteria: two or more values meeting or exceeding the following: fasting: 95 mg/dl, 1 h: 180 mg/dl, 2 h: 155 mg/dl, 3 h: 140 mg/dl) [25]. The patients' characteristics of GDM and control women are reported in Table 1. A venous blood sample was taken from all women between 17 and 41 weeks of gestational age, and none of them were taking insulin when the auto-antibodies were determined. Serum was separated by centrifugation, and kept at -20°C. Anti- GAD65, anti-IA2 and IAA, were determined by Radioligand Assay (Medipan GMBH-Berlin, Germany). Highly purified human recombinant antigens labeled with ^125^I was used to identify anti-GAD65 and anti-IA2. The cut-off limit for positivity was 0.9 and 0.75 U/ml for anti-GAD65 and anti-IA2, respectively. Highly purified human ^125^I-(A14) mono-iodinated insulin was used for the IAA determination. The cut-off limit for its positivity was 1 U/ml.

**Table 1:** Clinical characteristics of patients with GDM and control subjects

	GDM group	Control group	P
N	62	56	
Age (years)	34.0 ± 4.4	32.5 ± 5.0	-
Parity	0.4 ± 0.7	0.5 ± 0.6	-
Gestational Age (weeks)	26.3 ± 4.6	25.5 ± 5.0	-
BMI (kg/m^2^)	24.1 ± 4.6	22.0 ± 2.7	0.008

Data are means ± SD

After diagnosis, GDM patients were instructed to follow a moderately hypocaloric regimen (30 Kcal/kg ideal body weight) and trained to self-monitor their blood glucose, according to the ADA's recommendations [25]. Their glycemic control and the ultrasound monitoring of foetal growth were followed in our Outpatient Centre until delivery. Insulin therapy was started if they reported 2-hours postprandial glycemic levels ≥ 120 mg/dl and/or fasting levels ≥ 95 mg/dl, according with ADA[25].

### Statistics

The prevalence of antibody-positive subjects in the GDM group was compared to those of the control group with a Chi-Square test. Statistical differences between continuous variables were analysed with a Student t-test. All analyses were performed using XLSTAT v 2007 software. For all analyses, *P *< 0.05 was considered to be statistically significant.

## Results

GDM patients differed from control patients only in Body Mass Index (BMI), significantly higher in GDM patients (24.1 ± 4.6 kg/m^2 ^vs 22.0 ± 2.7 kg/m^2^, P = 0.008).

The prevalence of DRAs in GDM patients and controls are shown in Table 2. On the whole, 38.8% (24 out of 62) of the GDM patients were positive for at least one antibody: anti-IA2 was present in 29.0% (18 out of 62) of our GDM patients vs. 7.1% (4 out of 56, P < 0.001), while IAA was found positive in 14.5% of them (9 out of 62) and absent in the control subjects (P < 0.001). Anti-GAD65 was also present in our GDM patients, with a prevalence of 3.2% (2 out of 62) while it was absent in the control group (P = NS). Five GDM patients (8%) in the whole group were positive for 2 antibodies, which were IAA + anti-IA2 for four of them, and IAA+ anti-GAD for one volunteer (1.6%).

**Table 2:** Prevalence of auto-antibodies in GDM patients and control subjects

Autoantibody	GDM group (N = 62) N (%)	Control group (N = 56) N (%)	P
Anti-GAD65	2 (3.2)	0 (0)	-
Anti -IA2	18 (29.0)	4 (7.1)	< 0.001
IAA	9 (14.5)	0 (0)	< 0.001
TOTAL	24 (38.8)	4 (7.1)	< 0.001
2 antibodies	5 (8)	0 (0)	0.005

Differences between auto-antibodies-positive and -negative GDM patients are shown in Table 3. No differences were shown in age, BMI, requirement of insulin therapy, and neonatal birth weight. Pre-gestational weight was significantly lower (57.78 ± 9.8 vs. 65.9 ± 17.3 *P *= 0.04) in auto-antibodies-positive patients.

**Table 3:** Characteristics of GDM auto-antibodies -positive and -negative GDM patients

	Ab- positive (N = 24)	Ab- negative (N = 38)	*P*
Mean age (yrs, mean ± SD)	34.7 ± 4.4	33.6 ± 4.5	0.36
Pre-gestational weight (Kg, mean ± SD)	57.78 ± 9.8	65.9 ± 17.3	0.04
BMI	22.7 ± 3.5	24.9 ± 5.1	0.09
Parity (N, mean ± SD)	0.28 ± 0.78	0.51 ± 0.74	0.27
Insulin therapy (N, %)	6 (25%)	6 (15.7%)	0.3
Birth weight (Kg)	3 ± 0.6	3.1 ± 0.6	0.6

## Discussion

Screening, diagnosis and treatment of GDM are very important in order to prevent related complications. Moreover, GDM identifies women who are at risk of developing diabetes later in life, usually Type 2 diabetes and less frequently Type 1 diabetes [20-22]. An early diagnosis of Type 1 diabetes is shown to preserve endogenous insulin secretion and to decrease the frequency of micro-vascular complications [26]. For these reasons, detecting during pregnancy the markers that identify individuals at risk of developing Type 1 diabetes in the future could be very important for public health in regions where there is a high prevalence of this disease. We focused our attention on previous reported high prevalence of GDM in a large group of Sardinian women [6] and the well-known high prevalence of Type 1 diabetes in Sardinia [7]. Our findings on DRAs in GDM women are very different from what has been reported with regard to Italy. Two Italian studies [18,19] reported a very low prevalence of all auto-antibodies in GDM patients. Specifically, anti-IA2 was completely absent in the patients screened in both studies, whereas anti-GAD was absent in one study [18] and present in 1.4% of GDM patients in the other [19]. IAA was tested only in one study [18], with a reported prevalence of 3%. The low antibody prevalence is likely to be related to the low incidence of Type 1 diabetes in the Northern Italy, which is one of the lowest in Europe. Thus, although little difference could be ascertained between our results and those of other Italian studies regarding the prevalence of GAD, the greatest difference regards the anti-IA2 and IAA values. Indeed, anti-IA2 was the most frequent antibody (present in 29% of our patients), while it was in fact absent in other Italian study groups. The different expression of DRAs in different geographic areas could be related to different genetic backgrounds, resulting in a different expression of the immune reactivity. Indeed, HLA genotypes appear to have a modifying influence on the expression of diabetes associated auto-antibodies. Anti-IA2 positively correlated with DQ8 and/or DR4/DQB1*0201 and was negatively associated with DR3/DQB1*0201 [27]. Similarly, IAAs and ICAs are found with a higher frequency in individuals positive for DR4 and DQ8, while anti- GAD65 is more commonly found in Type 1 diabetic patients with DR3 and or DQB1*0201 haplotypes [27]. It has been suggested that the DR3-associated anti GAD65 response is a marker of general autoimmunity, whereas the DR4-associated anti-IA2 response is a more specific marker of β-cell destruction [27]. However these suggestions seem to be in contrast with our higher prevalence of anti-IA2 and the more common Sardinian haplotype HLA DR3. Unfortunately no study which correlates genetic and autoimmune markers of Type 1 diabetes in the Sardinian population, has ever been conducted to date. Moreover, the only study carried out in schoolchildren and newborns did not show a higher frequency of any specific autoantibody [14].

It may be hypothesized that our DRAs – positive patients could have a condition known as LADA (Latent Autoimmune Diabetes in Adults). However, ICA and GADAs are also common in LADA, but both anti-IA2 and IAA are much less common in LADA than Type 1 diabetes [28].

Anti-IA2 is present in the majority of individuals with new onset Type 1 diabetes and, in the pre-diabetic phase of the disease, its appearance seems to be correlated with the rapid progression to overt Type 1 diabetes [24,29,30]. Anti-IA2 was also present in 7.1% of our control subjects, but this is in agreement with findings by Jarvela [18,21], who reported variable percents of positivity for all DRAs in the control group. Moreover, it must not be forgotten that these antibodies are first of all markers of Type 1 diabetes and that they can in theory be present in any individual, independently of the presence GDM, in particular in areas where there is a high prevalence of Type 1 diabetes. Nevertheless, the meaning of positivity of this antibody on GDM patients remains to be clarified because in two studies [20,21], the anti-IA2 did not represent an independent risk factor for the development of Type 1 diabetes postpartum, whereas anti-GAD and ICA did. However, when found positive together with anti-GAD, it increased the screening sensitivity of anti-GAD from 63% to 75% [20].

IAA often precedes other autoimmune markers [27], and this led to the hypothesis that insulin may be an auto-antigen in Type 1 diabetes, which may play a role early in the pathogenic process. For this antibody too, like the anti-IA2, it is unknown whether its presence in GDM could be useful, because in the Finnish study [21], none of the women with previous GDM who developed Type 1 diabetes tested positive for IAA.

It is interesting that though the BMI of GDM patients was higher than controls, as would be expected since being overweight is a well known risk factor for GDM [25], the pre-gestational weight was significantly lower in DRAs-positive patients. This finding is in line with other published studies [17], in that DRAs-positive patients are usually slim, and could be consistent with a future development of Type 1 diabetes rather than Type 2, since the latter is usually associated with obesity. In contrast, our DRAs-positives patients are not younger than the DRAs-negatives ones, as shown for the patients affected by GDM who develop Type 1 diabetes after pregnancy [21].

The use of insulin for the treatment of GDM, which reflects the severity of the disease, was shown to be an independent risk factor for the occurrence of postpartum Type 1 diabetes [20,21]. We could not show a significant difference in the need for insulin therapy of DRAs-positive patients, as could be hypothesized if an immune-mediated destruction of pancreatic β-cells had occurred. However, a difference exists (25% vs. 15% in Ab-negative patients) which should be confirmed in a larger group of patients, in order to have statistical significance. Likewise, a more severe disease could not be shown in the group characterized by a greater neonatal birth weight, although this could signify that our protocol of monitoring and therapy was effective.

Parity was chosen as a comparison parameter, since other studies [20,24] found that women with one or more previous pregnancies had a higher risk for both Type 1 and Type 2 diabetes, but no difference was found in parity between DRas-positive and DRas-negative patients.

## Conclusion

Our findings are in contrast with the very low prevalence of all antibodies reported in Italy, and could be consistent with the peculiarity of the genetic and immunological features of the Sardinian population. If confirmed, they could indicate that a large proportion of GDM patients in Sardinia have an autoimmune origin, in accordance with the high prevalence of Type 1 diabetes.

The screening for auto-antibodies in pregnant women with GDM in populations at high risk for Type 1 diabetes has been recommended to allow an early diagnosis and, consequently, an appropriate therapy [20].

However, our results need further, extensive follow-up studies on a larger obstetric population, to verify whether or not our DRAs-positive patients are really at risk of developing Type 1 diabetes. A genetic characterization of DRAs-positive GDM patients would be also opportune.

## Competing interests

The authors declare that they have no competing interests.

## Authors' contributions

CM conceived of the study, its design and coordination and draft the manuscript, MO carried out the Radioligand Assays, EP collected samples and results, RB helped to draft the manuscript, CM performed the statistical analysis, SS, MM, NG and PZ participated in patients recruitment and data collecting, AMP directed laboratory procedures, and GBM made substantial contribution to conception and design and was ultimately responsible for this work. All authors read and approved the final manuscript.
